# Expression characteristic of *4Ig B7-H3* and *2Ig B7-H3* in acute myeloid leukemia

**DOI:** 10.1080/21655979.2021.2001182

**Published:** 2021-12-11

**Authors:** Wei Zhang, Lingyi Zhang, Jun Qian, Jiang Lin, Qiaoyun Chen, Qian Yuan, Jingdong Zhou, Tingjuan Zhang, Jinning Shi, Hong Zhou

**Affiliations:** a Department of Hematology, The Affiliated Jiangning Hospital with Nanjing Medical University, Nanjing, China; b Laboratory Center, The Affiliated People’s Hospital of Jiangsu University, Zhenjiang, China; c School of Medicine, Jiangsu University, Zhenjiang, China; d Department of Hematology, The Affiliated People’s Hospital of Jiangsu University, Zhenjiang, China

**Keywords:** *4Ig B7-H3*, *2Ig B7-H3*, acute myeloid leukemia, prognosis

## Abstract

*4IgB7-H3* (*4Ig*) and *2IgB7-H3* (*2Ig*) expression characteristics in acute myeloid leukemia (AML) remain unknown. This study investigated mRNA and membrane protein expression of two *B7-H3* isoforms in AML cell lines and de novo patients by using RT-PCR and flow cytometry, and analyzed the *B7-H3* promoter methylation state by utilizing RQ-MSP. *4Ig* was the dominant isoform. *2Ig* mRNA expression rate and abundance were elevated in AML in comparison with controls (*P* = 0.000 and 0.000), while no significant difference in *4Ig* (*P* = 0.802, *P* = 0.398). Membrane protein levels of *B7-H3* isoforms in AML was higher than controls, detected by total *B7-H3* expression rate (*P* = 0.002), total *B7-H3* mean fluorescence intensity (MFI) ratio of blast cells and lymphocytes (MFI ratio) (*P* = 0.000), and *4Ig B7-H3* MFI ratio (*P* = 0.005). Compared with *2Ig*^low^ group, *2Ig*^high^ patients had older age, lower *NPM1* mutation, higher *FLT3-ITD* mutation, and declining complete remission (CR) rates (*P* = 0.026, 0.012, 0.047, and 0.028). In *B7-H3*^high^ group, there was a trend toward older age, M4 and M5, worse karyotype, and lower CR rates, although with no marked difference (*P* > 0.05). The overall survival (OS) of *2Ig*^high^ and *B7-H3*^high^ groups were shorter than that of *2Ig*^low^ and *B7-H3*^low^ groups in the whole and non-M3 AML cohorts (*P* = 0.006 and 0.046; *P* = 0.003 and 0.032). Besides, an unmethylated *B7-H3* promoter was identified. In conclusion, *2Ig* mRNA and total *B7-H3* membrane protein tended to have potential diagnostic value for AML. Specifically, high *2Ig* mRNA and total *B7-H3* membrane protein expression indicate worse OS. *4Ig* and *2Ig* expression are methylation-independent.

## Introduction

Acute myeloid leukemia (AML) is a heterogeneous hematologic malignancy characterized by abnormal growth and differentiation of hematopoietic stem cells. In AML, immature myeloid precursors accumulate in bone marrow (BM), peripheral blood, and/or other tissues at the expense of normal terminally differentiated counterparts [1]. With constant advances in the study on AML genetic and epigenetic characterization, a variety of new targeted drugs have emerged and been established to tackle the pathophysiology within individual AML subsets [2]. Although the new therapies had improved the AML patients outcomes, these treatment options pose some new challenges for physicians to treat unfit populations, and the refractory/relapsed patients [3]. The personalized treatment of AML is a promising strategy focusing on the targets in survival and propagation pathways of AML cells and combination therapies [4]. Hence, it is important to identify new valuable biomarkers, which can contribute to recognize the poor prognosis of AML patients and to distinguish the biological drivers before the individual treatment of AML.

*B7-H3* (CD276), a part of the B7 immunoregulatory family, involves two isoforms *4IgB7-H3* (*4Ig*) and *2IgB7-H3* (*2Ig*) in humans. The two isoforms have similar structures, with extracellular immunoglobulin domain IgV-IgC for *2Ig* and IgV-IgC-IgV-IgC for *4Ig*, and *4Ig* is the main isoform expressed in malignant cells [5,6]. *B7-H3* exon duplication generates a conserved region in the first IgC domain of *4Ig*, which could disable *4Ig* from releasing soluble form, whilst *2Ig* produced both membrane and soluble forms [7]. *B7-H3* mRNA is ubiquitously expressed in non-lymphoid and lymphoid organs but with limited protein expression, suggesting the presence of post-transcriptional control mechanisms [8,9]. *B7-H3* had both co-stimulatory and co-inhibitory immunoregulatory functions. It was reported that *2Ig* could increase the proliferation of T cells and the production of IL-2, IFN-γ, while *4Ig* could decrease cytokine production and T cell proliferation [7]. In many solid tumors and AML patients, aberrant *B7-H3* protein overexpression has been indicated and linked to poor prognosis [10,11], suggesting that *B7-H3* may have the capability in becoming a therapeutic target. There was a relationship concerning alternative splicing events and DNA methylation across ten human solid tumor types through genome-wide analysis [12]. A CpG Island located in *B7-H3* promoter region was revealed using the UCSC database. However, *B7-H3* isoforms expression characteristics and clinical significance and *B7-H3* methylation levels in AML have not yet been elucidated.

This study hypothesized that the two *B7-H3* isoforms had different expression state, prognostic significance, and methylation levels in AML. First, the mRNA and membrane protein expression of *4Ig* and *2Ig* in AML cell lines and de novo patients was investigated. Then the prognostic correlation analysis was carried out. Additionally, this study analyzed the association between the methylation state of *B7-H3* promoter and the two isoforms mRNA expression. This research aimed at valuing the diagnosis and prognostic risk estimation for *B7-H3* isoforms in AML, and providing the foundation for further study on function and regulatory mechanism.

## Materials and methods

### Cell lines and cell culture

Human acute promyelocytic leukemia cell lines (NB4, HL-60), human acute monocytic leukemia cell lines (THP-1, U937, and SHI-1), human acute erythroleukemia cell line (HEL) and human chronic myeloid leukemia transformed to erythroleukemia cell line (K562) were donated by the First Affiliated Hospital of Soochow University. Human myelodysplastic syndrome transformed to acute monocytic leukemia cell line (SKM-1) was donated by the First Affiliated Hospital of Zhejiang University. The cell lines were cultured in RPMI-1640 (Thermo Fisher Scientific, Shanghai, China) medium comprising 10% heat-inactivated fetal bovine serum (Sciencell, Carlsbad, US), 100 U/mL penicillin and 100 μg/mL streptomycin (Harbin Pharmaceutical Group, Heilongjiang, China), at 37° in a 5% CO_2_ incubator. AML cell lines in the logarithmic growth phase were collected for the subsequent assays.

### Patients characteristics

This research was approved by the Institutional Ethics Committee of both the Affiliated Jiangning Hospital with Nanjing Medical University and the Affiliated People’s Hospital of Jiangsu University. With written informed consent provided by each subjects, the BM samples were collected from 68 de novo AML patients and 32 healthy donors, who were enrolled in the Department of Hematology in the Affiliated Jiangning Hospital with Nanjing Medical University and the Affiliated People’s Hospital of Jiangsu University, respectively. Healthy donors enrolled in this study were qualified on physical examinations and BM inspections, and had no medical history such as blood, heart, liver, kidney, digestive, neuropsychiatric system diseases and metabolic abnormalities; there were 17 males and 15 females, with a median age of 39.5 (19–58 years) years. AML patients were diagnosed in accordance with the 2008 World Health Organization diagnostic criteria for AML [13]. The following subjects were excluded: patients with other hematopoietic diseases except AML, and AML patients accompanied with other solid tumors. The AML patients were treated with combined chemotherapy containing induction therapy and consolidation and maintenance therapy subsequently. Induction therapy for non-M3 AML (non-acute promyelocytic leukemia) patients consisted of singular or doubled courses of daunorubicin or idarubicin in combination with cytarabine. Consolidation therapy consisted of intermediate-dose or high-dose cytarabine, mitoxantrone combined with cytarabine and homoharringtonine in combination with cytarabine. For AML-M3 patients, the induction therapy consists of all-trans retinoic acid (ATRA) or in combination with arsenic acid, and chemotherapy, such as daunorubicin and cytarabine. Maintenance therapy consisted of ATRA, mercaptopurine, and methotrexate for two or more years [14]. The drug selection and dose of the above combined chemotherapy are based on NCCN Clinical Practice Guidelines in Oncology: Acute Myeloid Leukemia [15,16]. The clinical appearances and laboratory characteristics of AML patients were collected and analyzed.

### RT-PCR assay

Bone marrow mononuclear cells (BMMNCs) was gained from BM samples of de novo AML patients and healthy donors using Lymphocyte Separation Medium (TBD Sciences, Tianjin, China) in the density gradient centrifugation method. Total RNA of AML cell lines and BMMNCs was extracted utilizing the Trizol reagent (Invitrogen, Carlsbad, CA, US). Then, the total RNA was reverse transcribed into cDNA according to the manufacturer’s instructions. Table 1 illustrates *B7-H3* isoforms primer sequences. The reaction system was comprised of 1 μM primers, 12.5 μM 2× Vazyme LAmp Master Mix (Vazyme, Nanking, China) and 20 ng cDNA. The PCR conditions are listed as so: 5 min in 94°C; 35 or 28 cycles (primer1-4: 35 cycles, *β-actin*: 28 cycles) for 30 sec at 94°C, 30 sec in 56–69°C (primer1 56°C, primer2 and primer4 69°C, primer3 66°C, *β-actin* 59°C), 30 sec at 72°C (primer1, primer2, and *β-actin*) or for 90 sec (primer3 and primer4); and finally 72°C for 10 min. Each assay was carried out with the positive and negative controls and was in triplicate. The mRNA expression rate was calculated by the ratio of the number of cases expressing *4Ig* or *2Ig* in total numbers of tested cases. And the expression abundance, relative expression level toward reference gene *β-actin*, was calculated by the ratio of gray values of *4Ig* or *2Ig* to that of *β-actin* [17].

**Table 1.** Primer sequences for *B7-H3* isoforms

Primers	Products	Sequences (5ʹ-3ʹ)
Primer 1	*B7-H3* (134bp) [32]	F:5ʹ-CTCTGCCTTCTCACCTCTTTG-3ʹ R:5ʹ-CCTTGAGGGAGGAACTTTATC-3’
Primer 2	*4Ig B7-H3* (380bp) [25]	F:5ʹ-CTCACGAAGCAGGTGAAGCTGCC-3ʹ R:5ʹ-ACCTACAGCTGCTGGTGCGCAA-3’
Primer 3	*B7-H3* isoforms (1444bp and 790bp) [5]	F:5ʹ-CACAGGAAGATGCTGCGTCG-3ʹ R:5ʹ-CAATGAGACAGACAGACAGC-3’
Primer 4	*B7-H3* isoforms (1572bp and 918bp) [6]	F:5ʹ-CAGCCCTGGCATGGGTGTGCAT-3ʹ R:5ʹ-CCATCATCTTCTTTGCTGTCAGAG-3’
Primer 5	*β-actin* (205bp) [33]	F:5ʹ-TGACGTGGACATCCGCAAAG-3ʹ R:5ʹ-CTGGAAGGTGGACAGCGAGG-3’

### Flow cytometry (FCM) assay

The AML cell lines and clinical BM samples were resuspended in precooled phosphate-buffered saline (PBS) to obtain single-cell suspensions with the density of 1 × 10^6^ cells/mL. Subsequently, antibodies were used to stain the suspensions for 30 min on ice. After triplicate washes in PBS, a flow cytometry system (Beckmen Counter, Miami, FL, US) through the Kaluza software (Beckmen Counter, Miami, FL, US) was used to analyze the cells collecting at least 10,000 events. *B7-H3*-specific monoclonal antibodies PE-mouse anti-human CD276 (*B7-H3*) (clone 2E6) and PE-mouse anti-human CD276 (*B7-H3*) (clone 3H4), together with PE-IgG1 murine isotype control were purchased from Suzhou Bright Scistar Biotechnology Company. The antibody 2E6 could recognize both *4Ig* and *2Ig*, while the antibody 3H4 specifically bonded *4Ig*. Other monoclonal antibodies used for distinguishing the AML immunophenotype were as follows: CD45, CD13, CD33, CD34, CD117, HLA-DR, CD15, CD14, CD64, CD56, CD41, CD61, CD3, CD4, CD8, CD5, CD10, CD19, CD20, CD22, et al. (Beckmen Counter, Miami, FL, US). The membrane protein expression rate and mean fluorescence intensity (MFI) was assessed, and the MFI ratio of blast cells and lymphocytes was calculated [18].

### Real-time quantitative methylation-specific PCR (RQ-MSP) assay

The genomic DNA from AML cell lines and BMMNCs of clinical samples was separated with the use of a genomic DNA purification kit (Gentra, Minneapolis, MN, US), and chemically adjusted with CpGenome™ DNA Modification Kit (Chemicon, Ternecula, Canada). Methylated *B7-H3* promoter (M-*B7-H3*) primer sequences were as follows: 5ʹ- GAGTTTTAGAGTCGGCGC-3ʹ (forward) and 5ʹ-AAACGAAAACGTACGAACCT-3ʹ (reverse) with 120bp PCR products, while for the unmethylated *B7-H3* promoter (U-*B7-H3*) were 5ʹ-TTTGAGTTTTAGAGTTGGTGT-3ʹ (forward) and 5ʹ-CCTAAACAAAAACATACAAACCT-3ʹ (reverse) with 120bp PCR products. SYBR Premix Ex Taq II (TaKaRa, Tokyo, Japan) was employed to RQ-MSP reaction, and the whole system was operated on a 7500 Thermo Cycler (Applied Biosystems, CA, US). RQ-MSP conditions were as follows: 30 sec at 95°C; 40 cycles for 5 sec at 95°C, 60°C for 30 sec, 30 sec at 72°C; and finally 75°C for 30 sec. Each assay contained positive and negative controls. The normalized ratio (N_U-*B7-H3*_) was implicated in unmethylation levels of *B7-H3* promoter assessment. N_U-*B7-H3*_ was calculated with the following formula: N_U-*B7-H3*_ = (E_U-*B7-H3*_) ^ΔCT U-*B7-H3* (control-sample)^ /(E*_ALU_*) ^ΔCT *ALU* (control-sample)^.

### Statistical analysis

Mann-Whitney’s U test was performed to compare continuous variables between two groups, while the χ2 test to compare categorical variables between two groups. The receiver operating characteristic curve (ROC) and area under the curve (AUC) was used to separate AML patients into high and low *B7-H3* isoforms expression groups. Kaplan**–**Meier and Cox regression analyses were used to evaluate *B7-H3* isoforms expression’s effect on overall survival (OS). *P* < 0.05 was regarded as statistically significant. The SPSS 20.0 software (SPSS, Chicago, IL) was adopted for the statistical analysis.

## Results

Aberrant *B7-H3* expression has been reported to indicate and be linked to poor prognosis in AML and many other tumors. However, the expression characteristics and clinical significance of *B7-H3* isoforms and *B7-H3* methylation levels were unclear. We found that the high *2Ig* mRNA and high total *B7-H3* membrane protein expression indicated shorter OS compared with the relative low expression groups, respectively. It suggested that the *2Ig* mRNA and total *B7-H3* membrane protein expression might have potential diagnostic value for AML, although they was not identified to be independent prognostic biomarkers in multivariate analysis. *4Ig* and *2Ig* expression are methylation-independent.

### *The mRNA expression of* B7-H3 *isoforms in AML cell lines*

**Figure 1.**
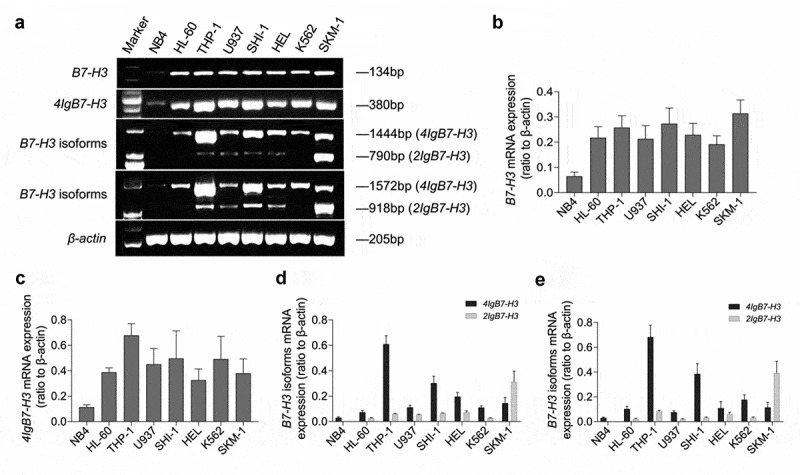
**The mRNA expression of *B7-H3* isoforms in 8 AML cell lines**. (a-e) RT-PCR was used to analyze mRNA expression with various *B7-H3*-specific primers. The ordinate in B-E represents gray values of *B7-H3, 4Ig B7-H3, B7-H3* isoforms (detected by *B7-H3*-specific primer 3), and *B7-H3* isoforms (detected by *B7-H3*-specific primer 4)/gray value of *β-actin*, respectively. The data is gained from no less than 3 individual experiments

We performed RT-PCR to detect the mRNA level of *4Ig* and *2Ig* in 8 AML cell lines (Figure 1). The results indicated that *B7-H3* was expressed in the whole-cell lines, showing the lowest expression in NB4. Except for SKM-1, *4Ig* was the dominant *B7-H3* isoform expressed in other 7 AML cell lines, especially in THP-1 and SHI-1. *2Ig* was weakly expressed in HL-60 and K562, but not expressed in NB4. Exclusively in SKM-1, the expression abundance of *2Ig* was remarkably higher than that of *4Ig* (*P* = 0.037 and 0.010, detected by *B7-H3*-specific primer 3 and primer 4, respectively).

**Figure 2.**
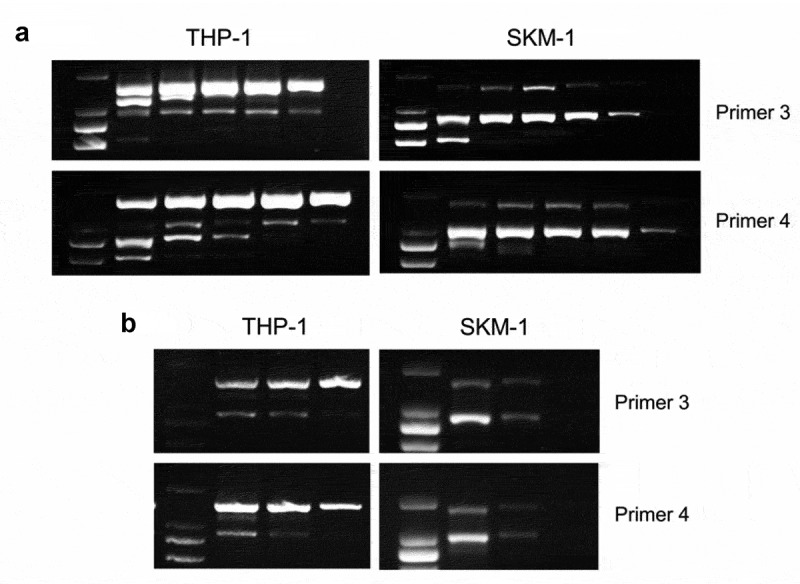
**RT-PCR analysis of THP-1 and SKM-1 with *B7-H3*-specific primers by changing PCR conditions**. (a) RT-PCR using *B7-H3*-specific primers in THP-1 and SKM-1 by increasing annealing temperature. The annealing temperature of primer 3 (up) and primer 4 (down) was 58.1°C, 61.3°C, 63.1°C, 66.4°C, 69°C and 59.9°C, 61.4°C, 62.5°C, 64.8°C, 68.9°C from left to right lanes, respectively. (b) RT-PCR using *B7-H3*-specific primers in THP-1 and SKM-1 by reducing cycles. The cycles were 35, 33, and 30 cycles from left to right lanes. The data is gained from no less than 3 individual experiments

To determine if minor bands of *B7-H3* isoforms were due to PCR artifacts, we performed RT-PCR with two sets of *B7-H3*-specific primers by increasing annealing temperature and reducing cycles in THP-1 and SKM-1 cells, which expressed the strongest *4Ig* and *2Ig*, respectively. The results showed that both the large and the small bands were detected persistently, whenever the PCR conditions changed in THP-1 and SKM-1 (Figure 2). Moreover, the two amplified PCR products of THP-1 and SKM-1 obtained using *B7-H3*-specific primer 3 were sent for DNA sequencing. The nucleic acid sequences of the large and minor band in two cell lines were consistent with that of *4Ig* and *2Ig* isoforms in the NCBI database (data not shown).

### *The mRNA expression of* B7-H3 *isoforms in de novo AML patients and controls*

**Figure 3.**
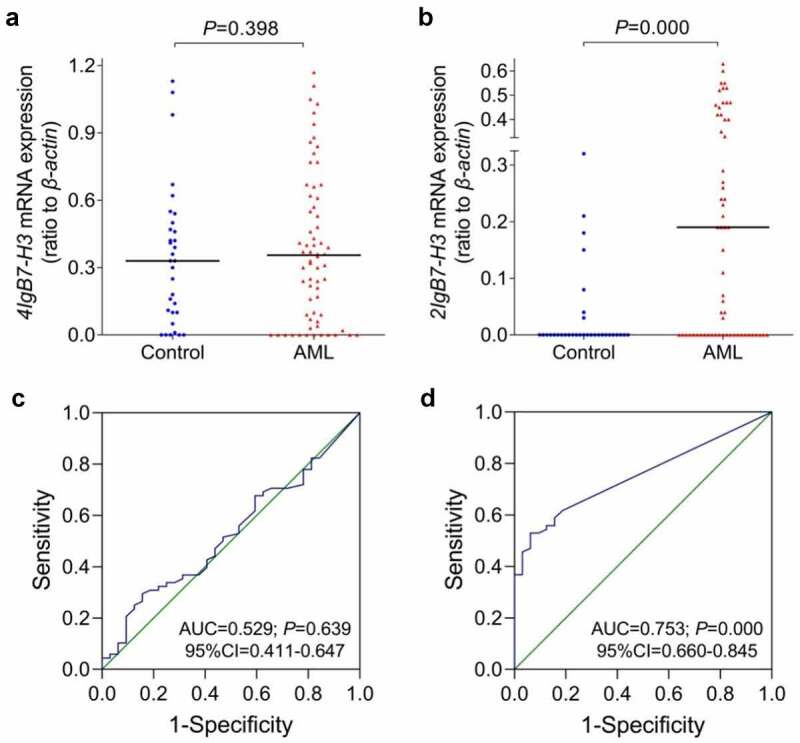
**The mRNA expression of *B7-H3* isoforms in de novo AML patients and controls**. RT-PCR with *B7-H3*-specific primer3 was used to detect mRNA expression of *B7-H3* isoforms in AML patients and controls followed by analysis by ROC curve. (a-b) The ordinate represents gray values of *4Ig B7-H3* and *2Ig B7-H3*/gray value of *β-actin*, respectively. (c-d) The ROC curve analysis of *4Ig B7-H3* and *2Ig B7-H3* mRNA expression. AUC, area under the ROC curve

The mRNA expression of *B7-H3* isoforms in 68 de novo AML patients and 32 healthy donors were analyzed using RT-PCR. The total *B7-H3* mRNA was widely expressed in the whole clinical samples (data not shown). Performing with *B7-H3*-specific primer 3, the mRNA expression rates of *4Ig* and *2Ig* were 82.4% (56/68) and 63.2% (43/68) in the AML group, while the expression rates were 84.4% (27/32) and 21.9% (7/32) in the control group, respectively. The expression abundance was calculated by the ratio of gray values of *4Ig* or *2Ig* to that of *β-actin* (Figures 3(a,b)). The results showed that neither the expression rate nor the expression abundance of *4Ig* had considerable discrepancies concerning the two groups (*P* = 0.802, *P* = 0.398). However, *2Ig* expression rate and abundance both increased in AML patients when in comparison with the controls (*P* = 0.000 and 0.000). The ROC was applied to differentiate AML patients from controls with mRNA expression of *B7-H3* isoforms (Figures 3(c,d)). It showed that *4Ig* could not distinguish all AML patients from controls (*P* = 0.639), while the *2Ig* mRNA expression could achieve it (*P* = 0.000). Similar phenomenon was also observed in the non-M3 AML patients compared with controls (data not shown). The cut off value of the *2Ig* expression level was 0.185, according to the maximum sum with sensitivity plus specificity in ROC analysis.

Subsequently, the whole AML cohort was separated into low (*2Ig*^low^) and high *2Ig* mRNA expression group (*2Ig*^high^), respectively (Table 2). Compared with the *2Ig*^low^ group, *2Ig*^high^ AML patients had the *older age* (*P* = 0.026), lower *NPM1* mutation (*P* = 0.012), higher *FLT3-ITD* mutation (*P* = 0.047), the declining complete remission (CR) rates after standard induction therapy for 2 cycles (*P* = 0.028), and a worse trend of karyotype classification (*P* = 0.056). No noteworthy variations in clinical manifestations and laboratory features were observed between the *2Ig*^low^ and *2Ig*^high^ groups, including sex, WBC, Hb, Plt, BM blasts, karyotype, and other common gene mutations (*P* > 0.05).

**Table 2.** Comparison of clinical manifestations and laboratory features between AML patients with low and high mRNA expression of *2Ig B7-H3* (*2Ig*)

Patient’s parameters	The whole AML patients		
	*2Ig*^low^ (n = 32)	*2Ig*^high^ (n = 36)	*P*
Sex, male/female	19/13	17/19	0.316
Median age, years (range)	55 (15–80)	66 (20–93)	0.026
Median WBC, ×10^9^ /L (range)	28.2 (0.8–136.1)	18.7 (0.8–207.5)	0.468
Median Hb, g/L (range)	88 (45–126)	86 (49–141)	0.672
Median Plt, ×10^9^ /L (range)	31 (3–192)	41 (5–382)	0.555
BM blasts, % (range)	50.0 (3.0*-94.0)	61.3 (16.5*-93.5)	0.414
FAB classification			0.371
M0	0 (0.0%)	2 (5.6%)	
M1	1 (3.1%)	2 (5.6%)	
M2	13 (40.6%)	15 (41.7%)	
M3	5 (15.6%)	2 (5.6%)	
M4	9 (28.1%)	6 (16.7%)	
M5	4 (12.5%)	8 (22.2%)	
M6	0 (0.0%)	1 (2.8%)	
Karyotype			0.361
Normal	12 (37.5%)	13 (36.1%)	
t(8;21)	2 (6.3%)	3 (8.3%)	
t(16;16)	2 (6.3%)	0 (0.0%)	
t(15;17)	5 (15.6%)	2 (5.6%)	
+8	1 (3.1%)	2 (5.6%)	
−5/5q-	0 (0.0%)	2 (5.6%)	
−7/7q-	0 (0.0%)	1 (2.8%)	
t(9;22)	0 (0.0%)	1 (2.8%)	
Complex	3 (9.4%)	6 (16.7%)	
Others	3 (9.4%)	5 (13.9%)	
Not available	4 (12.5%)	1 (2.8%)	
Karyotype classification			0.056
Favorable	9 (28.1%)	5 (13.9%)	
Intermediate	16 (50.0%)	19 (52.8%)	
Poor	3 (9.4%)	11 (30.6%)	
Not available	4 (12.5%)	1 (2.8%)	
Gene Mutation			
*NPM1* (+/-)	9/21	2/31	0.012
*CEBPA* (+/-)	8/22	5/28	0.259
*FLT3-ITD* (+/-)	3/27	10/23	0.047
*c-KIT* (+/-)	1/29	2/31	1.000
*N/K-RAS* (+/-)	4/26	6/27	0.857
*IDH1/2* (+/-)	2/28	5/28	0.504
*DNMT3A* (+/-)	3/27	3/30	1.000
*U2AF1* (+/-)	3/27	1/32	0.538
*SRSF2* (+/-)	3/27	2/31	0.912
*SETBP1* (+/-)	2/28	1/32	0.933
*TP53* (+/-)	1/29	3/30	0.675
CR (+/-)	19/12	12/23	0.028

*:Diagnosis was done in regards to the World Health Organization criteria for patients with low percentage blasts (< 20%) in BM. The cytogenetic aberrations t (15; 17) (q22; q12), were also detected in these patients. Abbreviations: AML = acute myeloid leukemia; WBC = white blood cells; Hb = hemoglobin; Plt = platelets; BM = bone marrow; CR = complete remission. *P* values were determined using χ^2^ test for the sex, karyotype, common gene mutations and CR rates after standard induction therapy for 2 cycles, and applying Mann-Whitney’s U test for the age, WBC, Hb, Plt and BM blasts.

### *The membrane protein expression of* B7-H3 *isoforms in AML cell lines*

**Figure 4.**
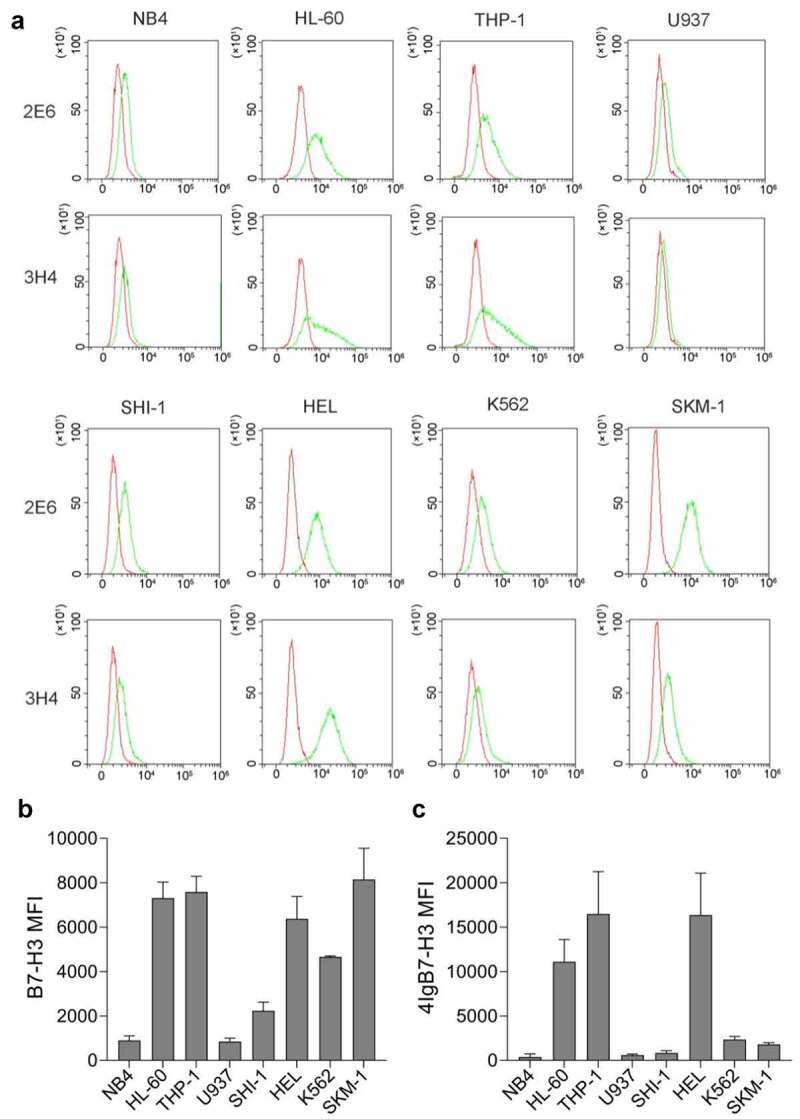
**B7-H3 isoforms in AML cell line membrane protein expression**. (a) The histograms revealed the expression of *B7-H3* isoforms, gating with isotype controls. The red and green lines in each histogram represent isotype control and *B7-H3* isoforms, respectively. The antibody 2E6 bound both *4Ig B7-H3* and *2Ig B7-H3*, while the antibody 3H4 specifically bound *4Ig B7-H3*. (b-c) The mean fluorescence intensity (MFI) of total *B7-H3* isoforms and *4Ig B7-H3* in AML cell lines. The data is gained from no less than 3 individual experiments

We selected two *B7-H3*-specific monoclonal antibodies and examined membrane protein expression of *B7-H3* isoforms in 8 AML cell lines by flow cytometry. 2E6 antibody could recognize both two types of *B7-H3* isoforms, while the antibody 3H4 specifically recognized *4Ig*, but not 2*Ig*. As shown in Figure 4, the majority of AML cell lines expressed total *B7-H3* isoforms detected by antibody 2E6, with the lowest expression abundance in NB4. The *4Ig* membrane protein detected by antibody 3H4 was also expressed in most AML cell lines, with no or low expression in NB4, U937, and SHI-1. Interestingly, the expression level of *4Ig* in K562 and SKM-1 was substantially lower, while total *B7-H3* isoforms expression was relatively higher. It was presumed that *2Ig* might be the main *B7-H3* isoform type for membrane protein expression in K562 and SKM-1.

### *The membrane protein expression of* B7-H3 *isoforms in de novo AML patients and controls*

**Figure 5.**
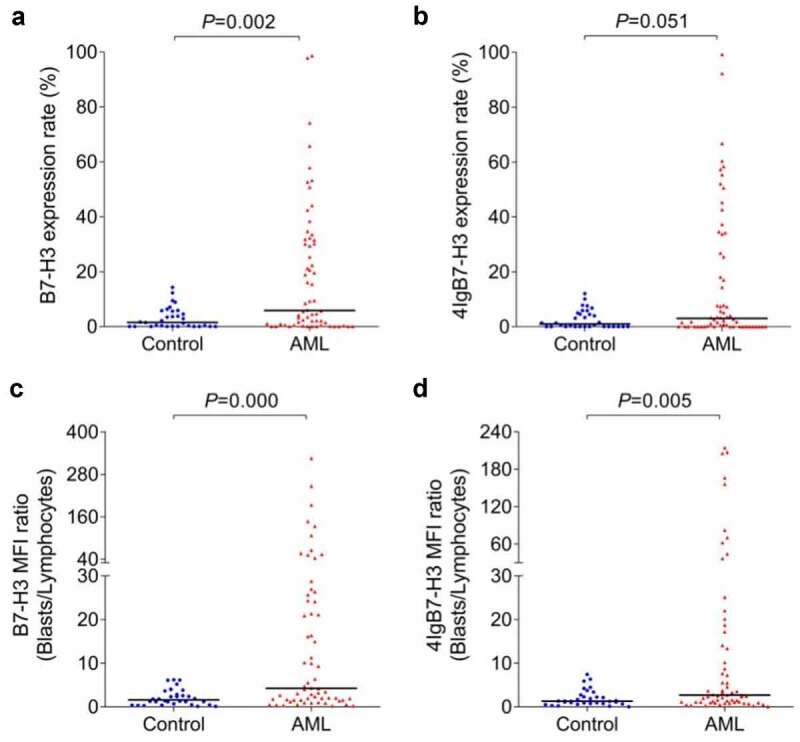
**The membrane protein expression of B7-H3 isoforms in de novo AML patients and controls**. (a-b) The expression rates of total *B7-H3* isoforms and *4Ig* on blast cells in AML patients and controls. (c-d) The mean fluorescence intensity ratio of blast cells and lymphocytes (MFI ratio) of total *B7-H3* isoforms and *4Ig B7-H3* in AML patients and controls

**Figure 6.**
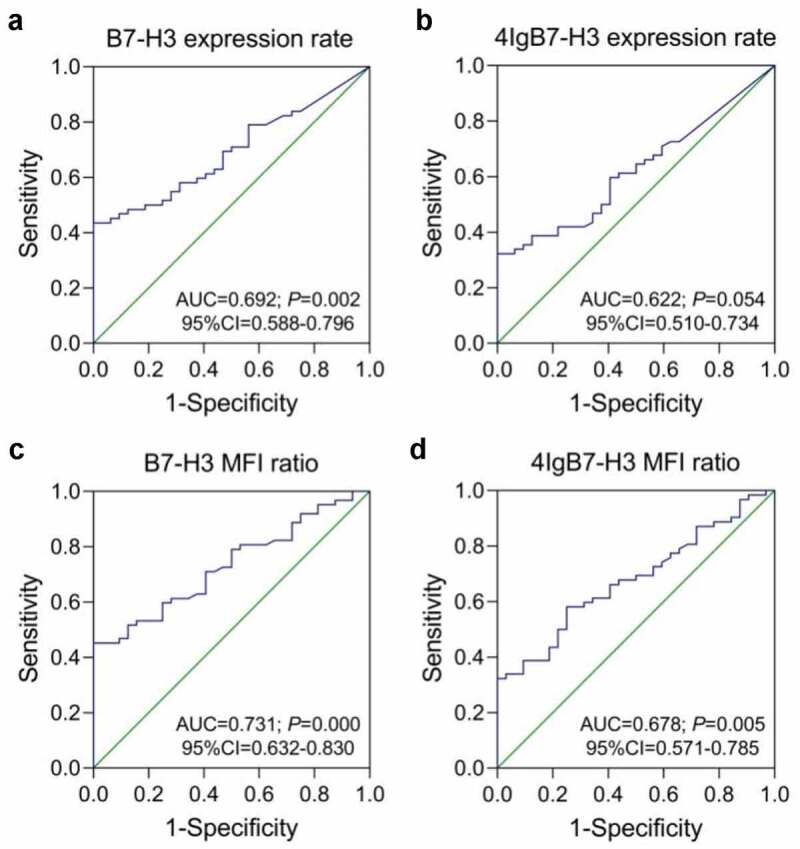
**The ROC curve analysis of B7-H3 isoforms membrane protein expression in de novo AML patients and controls**. (a-b) The expression rates of total *B7-H3* isoforms and *4Ig* on blast cells. (c-d) The mean fluorescence intensity ratio of blast cells and lymphocytes (MFI ratio) of total *B7-H3* isoforms and *4Ig B7-H3*. AUC, area under the ROC curve

The two *B7-H3*-specific monoclonal antibodies, 2E6 and 3H4, were also utilized for flow cytometry-based detection of membrane protein expression of *B7-H3* isoforms in 62 AML patients and 32 controls. There were six patients in the whole enrolled AML cohort had inadequate BM samples to detect membrane protein level of the two *B7-H3* isoforms. Compared with the controls, a significant increase was identified in the total *B7-H3* isoforms membrane protein expression rates on blast cells and MFI ratio of blast cells and lymphocytes in AML patients (*P* = 0.002 and 0.000). *4Ig* MFI ratio in the AML group was also higher when compared with the controls (*P* = 0.005), while *4Ig* expression rates had no noteworthy difference (*P* = 0.051) (Figure 5). Analyzing by ROC curves, the whole AML patients could be distinguished from controls through the total *B7-H3* expression rates (*P* = 0.002), total *B7-H3* MFI ratios (*P* = 0.000), and *4Ig* MFI ratios (*P* = 0.005) (Figure 6). Similar phenomenon was also observed in the non-M3 AML patients compared with controls (data not shown).

Furthermore, we selected the total *B7-H3* MFI ratios, which have the strongest discrimination, to analyze the *B7-H3* membrane protein expression level along with clinical manifestations and laboratory features in AML patients. The cutoff value was 6.265, determined by ROC analysis with the maximum of the Youden index. Then, the AML patients were separated into low (*B7-H3*^low^) and high membrane protein expression groups (*B7-H3*^high^) (Table 3). Although no noteworthy variances were found between the *B7-H3*^low^ and *B7-H3*^high^ groups in common clinical and laboratory features (*P* > 0.05), several indicators tended to occur in the *B7-H3*^high^ group, including older age, FAB classification of M4 and M5, worse karyotype classification, and lower CR rates.

**Table 3.** Evaluation of clinical manifestations and laboratory features amongst AML patients with low and high membrane protein expression of total B7-H3 isoforms

Patient’s parameters	The whole AML patients		
	*B7-H3*^low^ (n = 34)	*B7-H3*^high^ (n = 28)	*P*
Sex, male/female	18/16	16/12	0.741
Median age, years (range)	54.5 (15–93)	66 (32–90)	0.077
Median WBC, ×10^9^ /L (range)	18.6 (0.8–207.5)	33.3 (0.8–158.7)	0.581
Median Hb, g/L (range)	83 (45–141)	88 (50–138)	0.445
Median Plt, ×10^9^ /L (range)	34 (5–192)	35 (3–382)	0.983
BM blasts, % (range)	50 (3.0*-94.0)	64 (16.5*-93.5)	0.471
FAB classification			0.345
M0	2 (5.9%)	0 (0.0%)	
M1	1 (2.9%)	2 (7.1%)	
M2	16 (47.1%)	9 (32.1%)	
M3	5 (14.7%)	2 (7.1%)	
M4	6 (17.6%)	8 (28.6%)	
M5	4 (11.8%)	6 (21.4%)	
M6	0 (0.0%)	1 (3.6%)	
Karyotype			0.419
Normal	14 (41.2%)	10 (35.7%)	
t(8;21)	2 (5.9%)	2 (7.1%)	
t(16;16)	2 (5.9%)	0 (0.0%)	
t(15;17)	5 (14.7%)	2 (7.1%)	
+8	1 (2.9%)	1 (3.6%)	
−5/5q-	1 (2.9%)	1 (3.6%)	
−7/7q-	0 (0.0%)	1 (3.6%)	
t(9;22)	1 (2.9%)	0 (0.0%)	
Complex	2 (5.9%)	7 (25.0%)	
Others	4 (11.8%)	1 (3.6%)	
Not available	2 (5.9%)	3 (10.7%)	
Karyotype classification			0.165
Favorable	9 (26.5%)	4 (14.3%)	
Intermediate	19 (55.9%)	12 (42.9%)	
Poor	4 (11.8%)	9 (32.1%)	
Not available	2 (5.9%)	3 (10.7%)	
Gene Mutation			
*NPM1* (+/-)	7/25	3/22	0.534
*CEBPA* (+/-)	8/24	3/22	0.370
*FLT3-ITD* (+/-)	4/28	7/18	0.257
*c-KIT* (+/-)	0/32	2/23	0.188
*N/K-RAS* (+/-)	6/26	3/22	0.743
*IDH1/2* (+/-)	5/27	1/24	0.325
*DNMT3A* (+/-)	4/28	2/23	0.909
*U2AF1* (+/-)	4/28	0/25	0.190
*SRSF2* (+/-)	2/30	3/22	0.772
*SETBP1* (+/-)	2/30	1/24	1.000
*TP53* (+/-)	2/30	2/23	1.000
CR (+/-)	20/14	11/17	0.126

*:Diagnosis was done in regards to the World Health Organization criteria for patients with low percentage blasts (< 20%) in BM. The cytogenetic aberrations t (15;17) (q22; q12), were also detected in these patients. Abbreviations: AML = acute myeloid leukemia; WBC = white blood cells; Hb = hemoglobin; Plt = platelets; BM = bone marrow; CR = complete remission. *P* values were determined using χ^2^ test for the sex, karyotype, common gene mutations and CR rates after standard induction therapy for 2 cycles, and utilizing Mann-Whitney’s U test for the age, WBC, Hb, Plt and BM blasts.

### *Correlation amid* B7-H3 *isoforms expression and clinical outcome in de novo AML patients*

**Figure 7.**
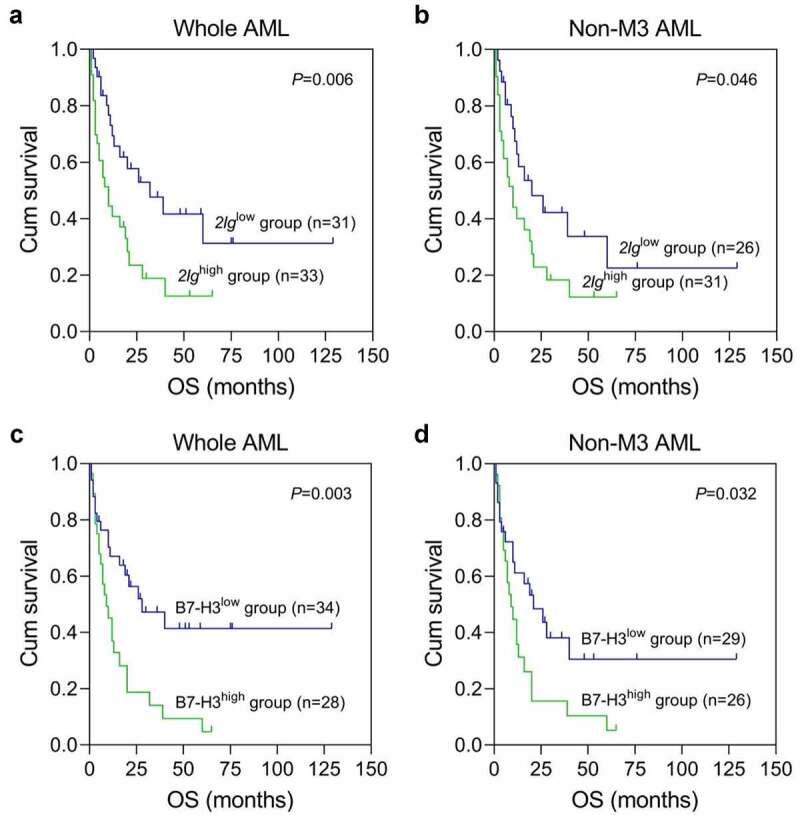
**The Kaplan–Meier analysis of B7-H3 isoforms expression in de novo AML patients**. (a-b) The influence of *2Ig B7-H3* mRNA expression on OS in the whole AML and non-M3 AML patients, respectively. (c-d) The influence of total *B7-H3* isoforms membrane protein expression on OS in the whole AML and non-M3 AML patients, respectively. Non-M3 AML, non-acute promyelocytic leukemia in AML; OS, overall survival

To explore prognostic significances of *2Ig* mRNA and total *B7-H3* membrane protein expression in AML patients, the survival figures were obtained from 64 de novo AML patients with a median follow-up time of 11.5 (1–129 months) months. Data from Kaplan–Meier analysis showed that OS in the *2Ig*^high^ group (estimated median OS: 10 and 10 months) was significantly shorter than that in the *2Ig*^low^ group (estimated median OS:32 and 20 months) in the whole and non-M3 AML patients (*P* = 0.006 and 0.046). Similarly, the *B7-H3*^high^ group (estimated median OS: 9 and 9 months) had worse OS compared with *B7-H3*^low^ group (estimated median OS: 28 and 21 months) in the whole and non-M3 AML cohorts, respectively (*P* = 0.003 and 0.032) (Figure 7).

The univariate analysis for OS in AML patients suggested that there were several potential poor prognostic factors in both whole AML patients and non-M3 AML patients, including age ≥ 60 years, WBC ≥ 30 × 10^9^/L, poor karyotypic classification, *NPM1* wild-type, *TP53* mutation, and without achieving CR after standard induction therapy for 2 cycles. High *2Ig* mRNA expression served as poor prognostic factor in the whole AML patients, while the high total *B7-H3* membrane protein as a poor biomarker in the non-M3 AML patients (Table 4). On account of *2Ig* mRNA expression and total *B7-H3* membrane protein were significant in one cohort for univariate analysis, they were included in multivariate analysis. The *TP53* mutation and no CR was an independent poor prognostic factor for OS both in the whole and non-M3 AML cohorts (*P* = 0.005 and 0.011 for *TP53* mutation, *P* = 0.000 and 0.001 for CR, respectively). Both high *2Ig* mRNA and *B7-H3* membrane protein expression reminded the trend to have a higher hazard ratio and shorter OS, although having no statistical difference (*P* > 0.05).

**Table 4.** Univariate and multivariate analysis of prognostic factors for overall survival in de novo AML patients

	Whole AML patients		Non-M3 AML patients	
	Hazard ratio (95%CI)	*P*	Hazard ratio (95%CI)	*P*
Univariate analysis				
Age(<60y/≥60y)	3.242 (1.663–6.308)	0.001	3.268 (1.590–6.718)	0.001
WBC(<30 × 10^9^/L /≥30 × 10^9^/L)	2.216 (1.187–4.139)	0.013	2.242 (1.171–4.293)	0.015
Karyotypic classifications				
(Favorable/Intermediate)	3.127 (1.071–9.130)	0.037	2.681 (0.628–11.452)	0.183
(Favorable/Poor)	8.836 (2.737–28.528)	0.000	7.604 (1.670–34.625)	0.009
(Favorable and intermediate/Poor)	3.705(1.775–7.736)	0.000	3.257 (1.555–6.821)	0.002
*2Ig B7-H3* mRNA expression (Low/High)	2.352 (1.245–4.444)	0.008	1.894 (0.989–3.625)	0.054
*B7-H3* membrane protein expression (Low/High)	1.776 (0.957–3.296)	0.069	1.975 (1.037–3.764)	0.038
*NPM1* mutation (Wild-type/Mutant)	0.321 (0.113–0.915)	0.033	0.261 (0.091–0.753)	0.013
*FLT3-ITD* mutation (Wild-type/Mutant)	1.973 (0.926–4.205)	0.078	1.956 (0.884–4.326)	0.098
*TP53* mutation (Wild-type/Mutant)	6.346 (2.042–19.716)	0.001	6.204 (1.972–19.516)	0.002
CR (No/Yes)	0.146 (0.068–0.314)	0.000	0.180 (0.082–0.395)	0.000
Multivariate				
*2Ig B7-H3* mRNA expression (Low/High)	1.581 (0.660–3.785)	0.304	1.724 (0.702–4.236)	0.235
Total *B7-H3* membrane protein expression (Low/High)	1.896 (0.858–4.191)	0.114	1.334 (0.557–3.193)	0.518
*TP53* mutation (Wild-type/Mutant)	9.291 (1.994–43.298)	0.005	8.027 (1.615–39.896)	0.011
CR (No/Yes)	0.124 (0.044–0.350)	0.000	0.162 (0.057–0.457)	0.001

Abbreviations: AML = acute myeloid leukemia; non-M3 AML = non-acute promyelocytic leukemia in AML; WBC = white blood cells; CR = complete remission.

### B7-H3 *methylation in AML cell lines, de novo AML patients and controls*

**Figure 8.**
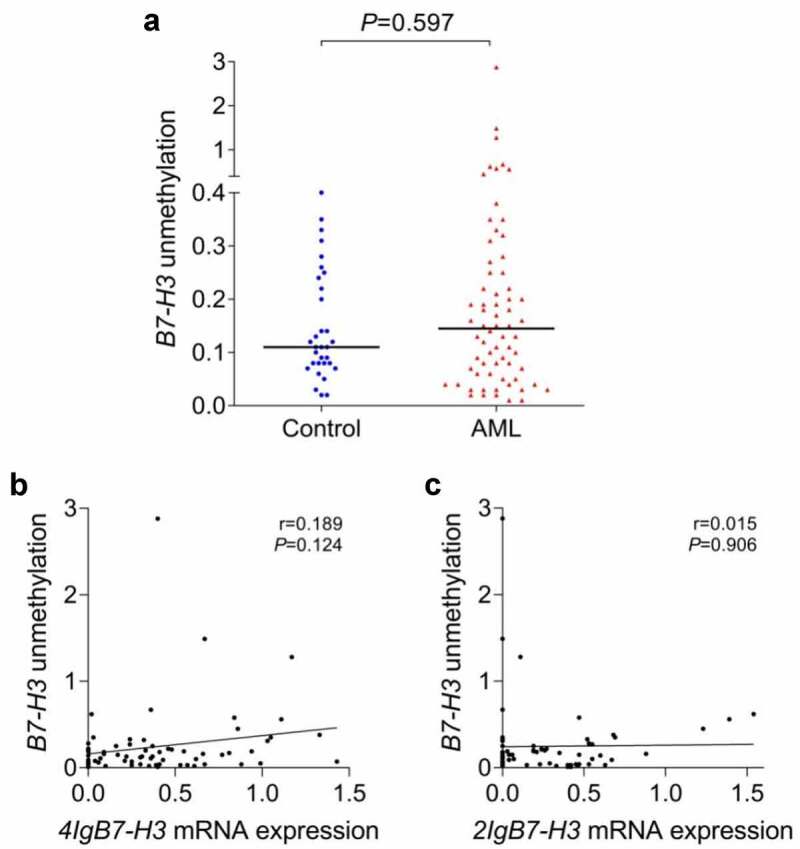
***B7-H3* unmethylation level and its correlation with the mRNA expression of *B7-H3* isoforms in de novo AML patients**. (a) *B7-H3* unmethylation level in AML patients and controls. (b-c) Correlation between *B7-H3* promoter unmethylation level and the mRNA expression of *4Ig B7-H3* and *2Ig B7-H3* in AML patients

RQ-MSP was designed to determine *B7-H3* promoter methylation state in 8 AML cell lines, 68 AML patients and 32 controls. In cell lines, the *B7-H3* methylation was detected in HL-60 and HEL, and partial methylation was found in U937 (data not shown). A majority of AML cell lines and clinical samples had the unmethylated state of *B7-H3* gene promoter, and no significant difference in *B7-H3* unmethylation level between AML patients and controls (*P* = 0.597, Figure 8(a)). Furthermore, we searched DiseaseMeth Databases (http://bioinfo.hrbmu.edu.cn/diseasemeth) and consistently confirmed that the methylation or low methylation level of the *B7-H3* promoter region had no marked difference between the AML group and controls (*P* = 0.6366). Besides, correlation analysis revealed that the unmethylation level of the *B7-H3* promoter had no significant relationship with either *4Ig* or *2Ig* mRNA expression (Figures 8(b,c)).

## Discussion

*B7-H3* gene was firstly identified in a human DC-derived cDNA library by Chapoval et al. [8]. The mouse *B7-H3* gene was described as a molecule consisting of two Ig-like domains (*2IgB7-H3, 2Ig*), while human *B7-H3* had the other isoform with four Ig-like domains (*4IgB7-H3, 4Ig*), resulting from gene duplication and differential splicing [5,19]. The two isoforms share identical exons, resulting in the same transmembrane and intracellular sequences [19]. In the present study, four sets of primers specific to *B7-H3* isoforms were selected to perform RT-PCR. Consistent with the previous studies, we found that the two *B7-H3* isoforms *4Ig* and *2Ig* were widely expressed in AML cell lines, de novo AML patients and controls, and they were persistently detected through RT-PCR by increasing annealing temperature and reducing cycles, followed by DNA sequencing confirmed. *4Ig* was the dominant type of isoform. Among 8 AML cell lines, SKM-1 had remarkably higher expression abundance of *2Ig* than that of *4Ig*, suggesting that the aberrant *2Ig* mRNA expression might participate in the progression of AML with myelodysplasia-related changes. Furthermore, the ROC curve analysis suggested that the *2Ig* mRNA expression level, but not 4*Ig*, could become a biomarker for diagnosing AML. This study firstly exhibited that both the mRNA expression rate and the expression abundance of *2Ig* were significantly elevated in de novo AML patients, and high *2Ig* mRNA expression indicated shorter OS in the whole and the non-M3 AML cohorts. The *2Ig* mRNA overexpression might harm the prognosis of AML patients, although it was no statistical differences in multivariate analysis. The independent poor prognostic factors for OS both in the whole and non-M3 AML cohorts were the *TP53* mutation and no CR after standard induction therapy for two cycles.

Aberrant protein overexpression of *B7-H3* has shown up in a wide range of solid cancer tissues, including the brain [20], lung [21], liver [22], pancreatic [23], and colorectal cancers [24]. It is linked to more advanced diseases and poor prognosis. In 134 acute leukemia patients, the *B7-H3* membrane protein expression was substantially superior in CD34+ cases, with a 44.8% positive rate, and predicted an unfavorable outcome in AML patients [11]. Another research reported that the membrane protein of *B7-H3* was expressed in blast cells in 30 of the 111 AML patients (27%), *B7-H3*-positive patients had prolonged event-free survival (*P* = 0.014) and improved overall survival tendency [18]. However, few studies have focused on individual *B7-H3* isoform’s protein expression characteristics. Wang et al. [25] generated two specific monoclonal antibodies recognizing *2Ig* and total *B7-H3* isoforms for an immunohistochemical test and found that *2Ig*, but not 4*Ig*, was specifically expressed in glioma. In this study, two *B7-H3*-specific antibodies 3H4 and 2E6, which specifically recognized *4Ig* and the total *B7-H3*, were selected for detecting membrane protein expression of *B7-H3* isoforms in AML cell lines, de novo AML patients and controls. It also confirmed that *4Ig* rather than *2Ig* was the dominant expression isoform in most AML cell lines and de novo AML patients. Interestingly, we found the SKM-1 cell line had a low membrane protein expression abundance of *4Ig*, while the total *B7-H3* expressed relatively higher. Similarly, with its mRNA expression state, *2Ig* might be the main *B7-H3* isoform type in SKM-1, and it was presumed again that *2Ig* might participate in the disease progression. The total *B7-H3* membrane protein and MFI ratio of blast cells and lymphocytes in de novo AML patients were higher than the controls. Perhaps because of the summarization of *2Ig* membrane protein into total *B7-H3* expression levels, total *B7-H3* expression rates and MFI ratios could preferably distinguish AML patients from controls. By ROC curve analysis, the total *B7-H3* MFI ratio, with the highest AUC value, might be a utility biomarker for AML diagnosis. Besides, the AML patients could be divided into low and high total *B7-H3* membrane protein expression groups with cut off value of 6.265, and the *B7-H3*^high^ patients accounted for 45.2% (28/62). In univariate analysis, the high *B7-H3* MFI ratio predicted a poor prognosis in the non-M3 AML cohort. However, the total *B7-H3* isoforms membrane protein expression level was not identified to be an independent prognostic biomarker in multivariate analysis.

Aberrant patterns of DNA promoter methylation is a common epigenetic alteration, which regulates several gene expressions and participates in leukemogenesis [26]. To explore the pathogenic mechanisms and biological characteristics of AML, it is recommended to include DNA methylation analysis in disease classification [27]. The promoter methylation at cg10586317 loci exhibited the negative association with *B7-H3* expression in lower-grade gliomas, which was analyzed through 15 methylation probes designed for *B7-H3* from Infinium HumanMethylation450 BeadChip [28]. *B7-H3* promoter methylation was also inversely correlated with *B7-H3* mRNA levels in 379 colorectal cancer samples, using TCGA data from cBioPortal (Spearman: −0.383) [29]. In the current study, we designed RQ-MSP to determine the promoter methylation state of *B7-H3* in AML cell lines, de novo AML patients and controls. Similarly, with the results from the DiseaseMeth database, the majority of AML cell lines and clinical samples had the *B7-H3* unmethylated state, and the unmethylation level showed no significant difference between AML patients and controls. Though, there was no substantial relationships between the unmethylation level of the *B7-H3* promoter and the mRNA expression of *B7-H3* isoforms. Collectively, it is indicated that *B7-H3* promoter methylation was not the main epigenetic mechanism regulating *B7-H3* isoforms expression in AML, differently from the previous results on some solid tumors. It had been stated that several miRNAs could regulate *B7-H3* gene expression in many solid tumors [30], and miR-506 could inhibit cell proliferation and invasion by suppress *B7-H3* expression in mantle cell lymphoma [31]. Thus, we should perform further studies to determine the epigenetic regulation mechanism of *4Ig* and *2Ig* expression in AML, including miRNAs. Besides, the gradually increased *B7-H3* mRNA levels were found in cases with gene copy number alterations (shallow deletions, diploid and copy number gains) in colorectal cancer samples from four studies queried with cBioPortal [29]. Therefore, whether the two *B7-H3* isoforms were genetically dysregulated in AML also requested further researches to assess.

This study has several limitations: Firstly, we initially collected a small dataset of AML patients to assess the expression characteristic of two *B7-H3* isoforms. Further studies with expanded cohorts are required to verify the results of two *B7-H3* isoforms expression state in AML. Secondly, we indirectly detected the membrane protein level of two *B7-H3* isoforms in flow cytometry by using two monoclonal antibodies, one of which could recognize both *4Ig* and *2Ig* and the other specifically bonded *4Ig*. Subsequent experiments should be validated by direct approaches to differentiate two isoforms on protein level, such as Western blot combined with flow cytometry. Although we found *B7-H3* isoforms expression were methylation-independent in AML patients, the main mechanism regulating the two isoforms expression were unclear. Therefore, the relative regulatory genes and signaling pathways of *B7-H3* isoforms should be screened through bioinformatics analysis, and then be validated by the *in vivo* and *in vitro* experiments.

## Conclusion

This study suggests that both overexpression of *2Ig* in mRNA level and total *B7-H3* in membrane protein level may tend to have potential diagnostic value in AML. The OS is worse in the patients with high *2Ig* mRNA or total *B7-H3* membrane protein expression, although they were not independent prognostic biomarkers. In addition, the expression of *B7-H3* isoforms is methylation-independent in AML, which is worth to perform further research focusing on epigenetic and genetical regulation in order to find new therapeutic targets.

## HIGHLIGHTS

- *2Ig B7-H3* mRNA expression was elevated in AML than that of controls.
- The OS of *2Ig* ^high^ group was shorter than *2Ig* ^low^ group in AML.
- Membrane protein expression of total *B7-H3* isoforms in AML was higher than controls.
- The worse OS was in *B7-H3*^high^ group in AML, compared with *B7-H3*^low^ group.
- *4Ig B7-H3* and *2Ig B7-H3* expression are methylation-independent in AML.
